# What can vertebrates tell us about segmentation?

**DOI:** 10.1186/2041-9139-5-24

**Published:** 2014-07-01

**Authors:** Anthony Graham, Thomas Butts, Andrew Lumsden, Clemens Kiecker

**Affiliations:** 1 MRC Centre for Developmental Neurobiology, King’s College London, London SE1 1UL, UK

**Keywords:** Segmentation, Metamerism, Somites, Rhombomeres, Pharyngeal arches, Vertebrates, Evolution

## Abstract

Segmentation is a feature of the body plans of a number of diverse animal groupings, including the annelids, arthropods and chordates. However, it has been unclear whether or not these different manifestations of segmentation are independently derived or have a common origin. Central to this issue is whether or not there are common developmental mechanisms that establish segmentation and the evolutionary origins of these processes. A fruitful way to address this issue is to consider how segmentation in vertebrates is directed. During vertebrate development three different segmental systems are established: the somites, the rhombomeres and the pharyngeal arches. In each an iteration of parts along the long axis is established. However, it is clear that the formation of the somites, rhombomeres or pharyngeal arches have little in common, and as such there is no single segmentation process. These different segmental systems also have distinct evolutionary histories, thus highlighting the fact that segmentation can and does evolve independently at multiple points. We conclude that the term segmentation indicates nothing more than a morphological description and that it implies no mechanistic similarity. Thus it is probable that segmentation has arisen repeatedly during animal evolution.

## Introduction

**Figure 1:**
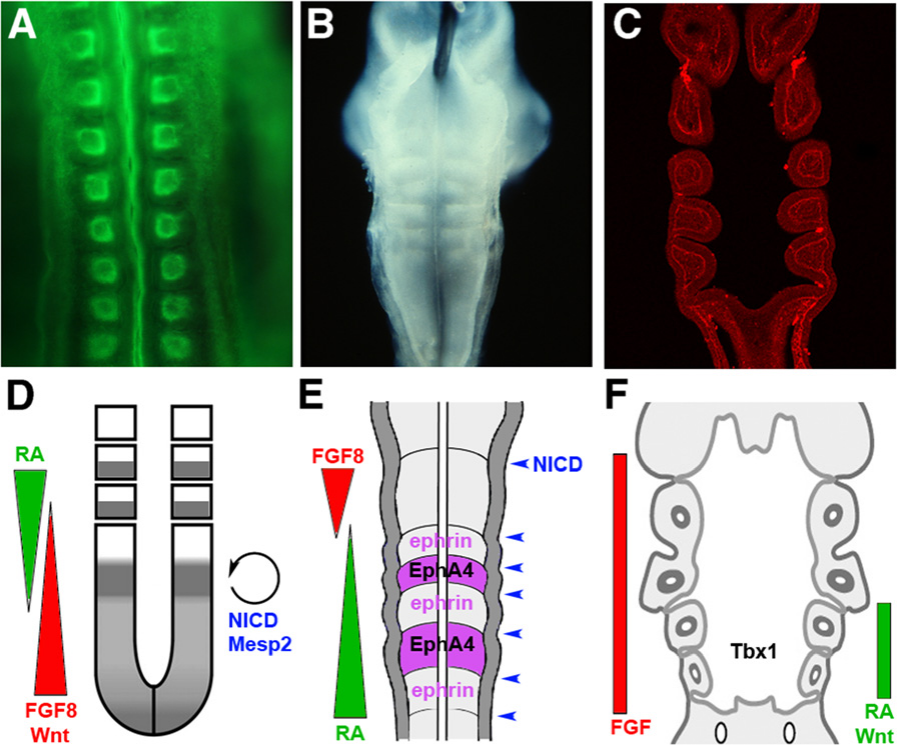
**Segmentation in vertebrates is governed by divergent molecular mechanisms: somites, rhombomeres and pharyngeal arches. (A)** A whole mount phalloidin staining of a stage 11 chick embryo viewed dorsally. The somites are readily visible as iterated blocks on either side of the neural tube. **(B)** An image of a dissected hindbrain region of chick embryo at 3 days of development. The roofplate has been removed to show the rhombomeres. **(C)** A longitudinal section through the pharyngeal region of a dogfish embryo. The embryo has been stained with an anti-laminin antibody. The pharyngeal segments are clearly shown. **(D)** Schematic diagram of somitogenesis. As the embryonic axis elongates, the presomitic mesoderm is patterned by opposing gradients of FGF8/Wnt activity (high posterior-low anterior) and retinoic acid (RA; low posterior-high anterior). Cycling expression of Notch pathway components (NICD = Notch Intracellular Domain) and of the transcription factor *Mesp2* acts as a pacemaker in this process. **(E)** Diagram of rhombomere (r) formation. R identity is regulated by a posteriorly increasing gradient of RA and by FGF8 signalling from the midbrain-hindbrain boundary. R boundary formation involves Notch activation (NICD) and they are maintained through cell sorting driven by alternating expression of EphA4 (in r3/5) and ephrin ligands (in r2, 4, 6). **(F)** The formation of all pharyngeal arches depends on FGF signalling, that of the posterior arches also on Wnt and RA signalling and on the transcription factor *Tbx1*. In all panels anterior is to the top.

Within the bilateria there are a number of clades, such as the arthropods, annelids and the chordates, which display the serial repetition of parts along the long body axis. These animals have been classified as being segmented [1]. There has, however, long been contention as to whether or not these instances of segmentation are homologous. A major problem that pervades this issue is how segmentation can be defined. A recent article by Hannibal and Patel, in this journal, makes the point that if we cannot understand exactly what is meant by segmentation and which structures are genuinely segmented then we have no hope of understanding how segmentation evolved and of clarifying the relationships between different types of segmentation [2]. These authors further suggest that any discussions of segmentation be clarified to explain exactly what is being studied and it is through this route that one might arrive at a better understanding of what segmentation is.To begin to understand segmentation in its different manifestations and to consider whether or not these are homologous processes across animal taxa we need to comprehend the segmentation process; that is, how segments arise and are allocated during development. With regard to this point, a study of diverse segmented structures in vertebrates is particularly useful. There are three clear distinct instances of segmentation being used as a developmental strategy in vertebrates and this affords one the opportunity to effectively and readily compare and contrast these processes (Figure 1). Moreover, we have a wealth of information about how these segmental systems are established. The first, and arguably the most well-known, is the formation of the somites from the paraxial mesoderm. The second is the subdivision of the hindbrain to generate rhombomeres. The last, and least discussed, is the formation of the pharyngeal arches, which are evident as serial bulges on the lateral surface of the embryonic head.

## Review

In this review, we consider how these segmentation processes are controlled and whether or not they have any relationship with each other. We also probe the evolutionary history of each of these segmental systems to gain insights into how they emerged during evolution and again we have a relatively solid knowledge of their origins. Additionally, we make no terminological distinction between ‘segmented’ and ‘metameric’ structures. All of the structures we discuss in vertebrates can be readily described as both segmented and metameric, and we make the argument that the only useful definition of such terms is morphological. Finally, we consider how the lessons we have learned from our consideration of segmentation in vertebrates feed into more general discussions of segmentation within the bilateria.

### Somites: segmentation of the paraxial mesoderm

The formation and regionalisation of the somites underpin the segmental organisation of the trunk of vertebrates [3,4]. Somites are blocks of mesoderm that bud off from the anterior end of the presomitic mesoderm as it retreats posteriorly and which will give rise to the vertebrae, skeletal muscle, connective tissue and regions of the dermis. The key events in the formation of the somites occur within the presomitic mesoderm and involve an interaction between a segmentation clock and opposing morphogen gradients. A component of the clock is the Notch pathway which acts to produce cycling gene expression such that a wave of expression, with a periodicity that matches somite formation, sweeps across the presomitic mesoderm [4]. The gradients take the form of a posterior to anterior gradient of Fgf8 [5,6] and nuclear b-catenin [7] and an anterior to posterior gradient of retinoic acid [8,9]. The intersection of these gradients defines the determination front, the point at which a cohort of cells at the same phase of oscillation is allocated to form a somite. This final step also involves the induction of *mesp2* expression, which suppresses Notch activity and thus facilitates the transition from cycling status to somite [10]. Thus, a significant facet of somitogenesis is that it is a drawn out process, largely concomitant with axis elongation, with many somites being formed over a protracted period. Central to this is the segmentation clock, which regulates the allocation of cells within the presomitic mesoderm to somites, and the continued posterior growth of the embryo.

However, although Notch signalling has been shown to oscillate in the presomitic mesoderm of all vertebrate models, its role in actually driving segmentation has been questioned. A recent study has even demonstrated that somites can form in the complete absence of the molecular clock indicating that a likely role of Notch may be in synchronising the segmentation clock rather than governing segmentation *per se*[11-13].

Another important feature of somites is that their segmental organisation is translated into the muscle segments of the body and the iterated vertebrae of the backbone. However, the positional relationship between the somites and the vertebrae is not a simple one to one correspondence but involves a resegmentation process [14,15]. In amniotes, the somites are subdivided into rostral and caudal halves with the rostral half sclerotome of each somite combining with the caudal half sclerotome of the immediately anterior somite to form each vertebra [16]. In zebrafish, studies have shown that while there is some evidence for resegmentation it is less strictly organised, with cells from the rostral or caudal half somites contributing to two consecutive vertebrae rather than exclusively to one [17].

This rostrocaudal organisation further impinges upon the neural crest cells that will form the peripheral ganglia and motor axons of the spinal nerves [18]. In amniotes, the caudal half sclerotome of each somite expresses a number of inhibitory molecules, including ephrins and semaphorins, while the neural crest and motor axon growth cones express the cognate receptors, and thus these populations are repelled by the caudal halves and only traverse the anterior half sclerotome [19-23]. This results in the secondarily derived segmentation of the peripheral nervous system and fixes the formation of the spinal nerves and ganglia in register with the vertebral column.

Finally, somites are also regionally distinct and those at different axial levels will generate vertebrae with different characteristics, such as cervical *versus* thoracic *versus* lumbar. Their anteroposterior identity is assigned within the presomitic mesoderm, prior to somite formation, and this involves differential *Hox* gene expression [24]. The number of somites formed varies greatly between different vertebrates; mice generate 65 somites, chicks 50, pythons greater than 500 and zebrafish about 31.

### Rhombomeres: segmentation of the hindbrain

Segmentation of the hindbrain is morphologically evident as a series of bulges and constrictions that subdivide this region of the brain and the number of segments, or rhombomeres (r), formed is largely invariant across the vertebrates [25-27]. The most anterior segment is r1, which lies just posterior of the mesencephalon, and the most posterior is continuous with the spinal cord. These structures define the ground plan upon which the development of the hindbrain is organised. Rhombomeres are, on some levels, serially homologous to each other but they also exhibit individual identities. The segments will generate similar cohorts of interneurons, but trigeminal motor neurons, for example, will only form in r2 and r3 and facial motor neurons only in r4 and r5 [25,28]. This two-segment periodicity also affects neurogenesis and axonal projection, which are initiated, and more advanced, in the even numbered rhombomeres (r2, r4 and r6) than in the odd numbered rhombomeres (r3 and r5) [29]. The even-numbered rhombomeres and the odd numbered also have distinct cell surface properties [30].

Rhombomeres differ from the somites in that they are not discrete separate structures formed by budding. Rather, they constitute a structural continuum that forms by the internal subdivision of a prespecified territory, the hindbrain primordium. This is underpinned by graded retinoid and wnt signalling, and the action of a range of different transcription factors that are needed to establish specific rhombomeres [31], including Krox-20 (r3/r5), Hnf1b/vHNF1 (r5/r6) and Hoxa1 (r5). The formation of the rhombomeres results in the emergence of lineage restricted compartments that are formed by cell segregation [32]. Prior to boundary formation cells can cross between proto-rhombomeric domains, but they cannot cross once a boundary has formed. Central to this phenomenon and indeed to the formation of the rhombomeres are cell-sorting events mediated by Eph/ephrin signalling [33]. The Eph receptors, such as *EphA4*, are expressed in r3 and r5 and the restriction of *Krox20* expression to these segments is likely to be involved in driving the expression of these receptors, while the ephrin ligands are expressed in r2, r4 and r6. This establishes bidirectional signalling interfaces between neighbouring rhombomeres which result in cell sorting such that like surrounds like. Thus cells within each rhombomere differentiate themselves from cells in their neighbouring segments, and cell-tight boundaries form. Finally, rhombomeres also differ from somites in that they do not simply form in anteroposterior sequence. The first rhombomeres to form are those in the central region of the hindbrain, r3 to r5, and this is then followed by the more anterior and posterior segments [34].

Once the segments have formed the individual identities of the rhombomeres become apparent and these are under the control of differential *Hox* gene expression. For example, r2 expresses only *Hoxa2*, while r4 expresses *Hoxa2*, *Hoxb2* and *Hoxb1* and r6 *Hoxa2*, *Hoxb2*, *Hoxa3* and *Hoxb3*. The differential Hox gene expression domains are in part directed by their differential transcriptional response to graded retinoic acid activity [35].

The segmentation of the hindbrain also has consequences for more peripheral tissues. A conserved feature of all vertebrates is the presence of three streams of neural crest emanating from the hindbrain. The most rostral is the trigeminal, which comprises neural crest cells from the midbrain and r1 and r2, the middle is the hyoid, which is primarily populated by neural crest cells from r4 with a small contribution from the flanking rhombomeres, and the most posterior is the post-otic which is composed of neural crest cells migrating from the caudal hindbrain [36,37]. These different streams come to populate distinct pharyngeal arches and they act to organise the sensorimotor innervation [36,38].

### Pharyngeal arches: segmentation of the pharynx

The pharyngeal arches are a series of bulges on the lateral surface of the head of vertebrate embryos. These structures are a key characteristic of the phylotypic stage and indeed lend that stage its name, the pharyngula. The development of these structures is complex as they comprise a number of disparate cell populations from all three germ layers [39]. Within each arch there is a mesodermal core, that will form muscle and endothelium and this is surrounded by neural crest cells, which will generate the skeletal and connective tissue components. These two mesenchymal populations are enveloped externally by ectoderm, which will generate epidermis and neurogenic placodes, and internally by the pharyngeal endoderm, which will produce the lining of the pharynx, taste buds and specialised structures such as the thyroid, thymus and parathyroids. Thus, the pharyngeal arches constitute an iterated series with each generating the same basic set of components.

An early key event in the development of the arches is the formation of outpocketings within the pharyngeal endoderm, the pharyngeal pouches [40,41]. Mutants that fail to segment the endoderm fail to form pharyngeal arches [42]. The pharyngeal pouches form at distinct positions along the anteroposterior axis and they will contact the overlying ectoderm, which invaginates to meet them, and form the pharyngeal clefts. The points of contact between the pouches and clefts define the anterior and posterior limits of the arches. Thus the neural crest and mesoderm migrate into pre-existing epithelial segments formed by segmentation of the endoderm.

The formation of the pharyngeal pouches requires signals from the surrounding mesodermal and neural tissues and it has been shown that Fgf function is necessary for the formation of all of the pouches [43]. There are, however, also significant differences between the development of the anterior and posterior pouches. First, the two most anterior pouches form at the same time, while the more posterior are generated sequentially [40,41]. The development of the posterior pouches is also under the control of distinct signalling pathways. It has been shown that the formation of the posterior but not the anterior pouches requires retinoic acid and wnt signalling from the adjacent lateral mesoderm [44-47]. *Tbx1* has also been identified as a key transcription factor for the generation of the posterior but not the anterior pharyngeal pouches [48], that drives proliferation within the endoderm. In *Tbx1* mutants the posterior pharyngeal pouches fail to form. Finally, it is also important to note that the number of pharyngeal pouches formed, and thus the corresponding number of arches, varies among the vertebrates, with a general trend towards reduction. Thus while lampreys form eight pouches and nine arches, many chondrichthyans and all actinopterygians form six pouches and seven arches, while amphibia form five pouches and six arches and amniotes four pouches and five arches.

Although segmentation of the endoderm provides the framework for pharyngeal development, interactions between the populations that contribute to the arches is also important for their full realisation. In particular, neural crest cells play a key role as they are the source of the skeletal elements that will underpin the later identity of the arches. In gnathostomes, the most anterior arch will form the jaws, the second the hyoid and the more posterior arches are either gill bearing in fish or subsumed into the larynx in amniotes. Again, the identity of the skeletal elements is dependent upon *Hox* genes.

### Segmentation in vertebrates is achieved through diverse, distinct unrelated mechanisms

With the formation of the somites, the subdivision of the hindbrain and the generation of the pharyngeal arches one can observe the iteration of parts along the long axis of the body. However, as is apparent from our consideration of how these processes are directed, they differ fundamentally from each other. There are few, if any, common mechanisms. Somites are generated sequentially over a long period using a clock and wave front mechanism acting within the presomitic mesoderm, which is to some extent replenished during axis extension. The rhombomeres form via internal subdivision of a specified region and over a very short period. The rhombomeres also do not emerge in an anteroposterior sequence with the first segments that are formed being r3, r4 and r5 [34]. Finally, the pharyngeal segments form through the outpocketing of the pharyngeal endoderm to form the pharyngeal pouches which contact the overlying ectoderm and thus delineate the anterior and posterior limits of the arches. There are, however, some superficial similarities such as the utilisation of some of same molecules, but these are often not employed in the same way. Thus while a posterior to anterior gradient of FGF is important for somitogenesis [5], this is not true of pharyngeal or hindbrain segmentation. In the pharynx, FGF activity is required for the formation the pouches themselves [43] and in the hindbrain FGF signalling has localised roles in the development of particular regions; early FGF is needed for r4 formation and FGF signalling from the isthmus impinges upon r1/r2 identity [49,50]. Another similarity lies in the identity of the segments being dependent on *Hox* genes, but this reflects the more general role of these genes in anteroposterior patterning of the body. *Hox* genes assign identity to segmented and unsegmented regions, such as the lateral plate mesoderm, alike.

Finally, it is also important to appreciate that these different segmented systems have different anatomical/functional outcomes and thus serve distinct functions. The segmentation of the paraxial mesoderm to generate individually packaged somites underpins the locomotory strategies of the vertebrates, resulting in the formation of separate bilateral bocks of muscle lying either side of an articulated backbone. This arrangement is essential for lateral undulatory locomotion of fish and many tetrapods. In contrast, the segmentation of the hindbrain generates subdivisions within a contiguous region which allows for seamless connections between the different hindbrain nuclei and ongoing connections, and for through traffic that connects higher brain centres with the spinal cord. This organisation of the hindbrain is vital to its function in co-ordinating respiratory activity, motor output and processing sensory information including that from the auditory/vestibular and lateral line systems. Lastly, the segmentation of the pharynx relates to its activities in feeding and respiration. The two most anterior pharyngeal segments of the gnathostomes will contribute to the jaw apparatus and the more posterior segments will form gills with an abundant vasculature and thus perform respiratory functions.

### The evolutionary origins of vertebrate segmentation

To further clarify the relationships between the vertebrate segmentation processes; somitogenesis, rhombomeric subdivisions of the hindbrain and pharyngeal arches, it is important to ask about their evolutionary origins.

Vertebrates are chordates and one defining feature of this phylum is the presence of segmented muscle blocks. We might therefore expect somitogenesis to be a shared characteristic of the chordates. Yet somites are lacking in urochordates and while they do form in cephalochordates this process seems to be somewhat distinct from that described in vertebrates [51]. The more anterior somites in amphioxus form as bilateral pairs by enterocoelus evagination of the wall of the archenteron while the posterior somites form by schizocoely, alternating between the left and right sides. It has also been shown that, while the very anterior somites are dependent upon FGF signalling, most of the other somites forming by enterocoely and those formed by schizocoely are FGF insensitive. However, the lineages leading to the extant representatives of the chordate subphyla diverged a very long time ago and thus ancestral characteristics may have been lost or obscured. Moving outside the chordates, and considering other deuterostomes one cannot find evidence for somites. Thus, it is reasonable to assume that somitogenesis evolved with the chordates but has undergone major modification in the different chordate lineages.

Gene expression studies in other chordates and in hemichordates have established that a region of the nervous system expressing anterior *Hox* genes, and thus homologous to the vertebrate hindbrain domain, exists in these groups [52]. There are, however, no indications that rhombomeres exist outside vertebrates. There is neither morphological nor molecular evidence to support segmentation of the nervous system in an analogous region. For example, amphioxus has a single *Krox20* gene but it is not expressed segmentally in the developing nervous system [53].

Pharyngeal segmentation is, however, relatively ancient. As with somites, pharyngeal gill slits are characteristic of the chordates and it is clear that the simple perforations of the pharynx seen in other chordates such as amphioxus are homologous to the endodermal segmentation of the vertebrate pharynx. The pharyngeal pouches of vertebrates express a *Pax-Six-Eya* regulatory network as does the pharyngeal endoderm in amphioxus [54,55]. Similarly, the amphioxus *Tbx1/10* gene is also expressed in the pharyngeal segments, mirroring the expression of *Tbx1* in vertebrates [56]. Recent results from the hemichordate Saccoglossus kowalevskii have shown that pharyngeal segmentation is likely to be a general feature of deuterostomes [57]. In this species, the formation of the gill pores by the endoderm is also associated with the expression of *Pax1/9*, *Eya* and *Six*. Furthermore, although echinoderms lack gill slits, this is likely to result from secondary loss as fossil evidence has shown that the earliest echinoderms were bilateral and did possess gill slits [58], which further indicates that pharyngeal segmentation is a characteristic of the deuterostomes.

**Figure 2:**
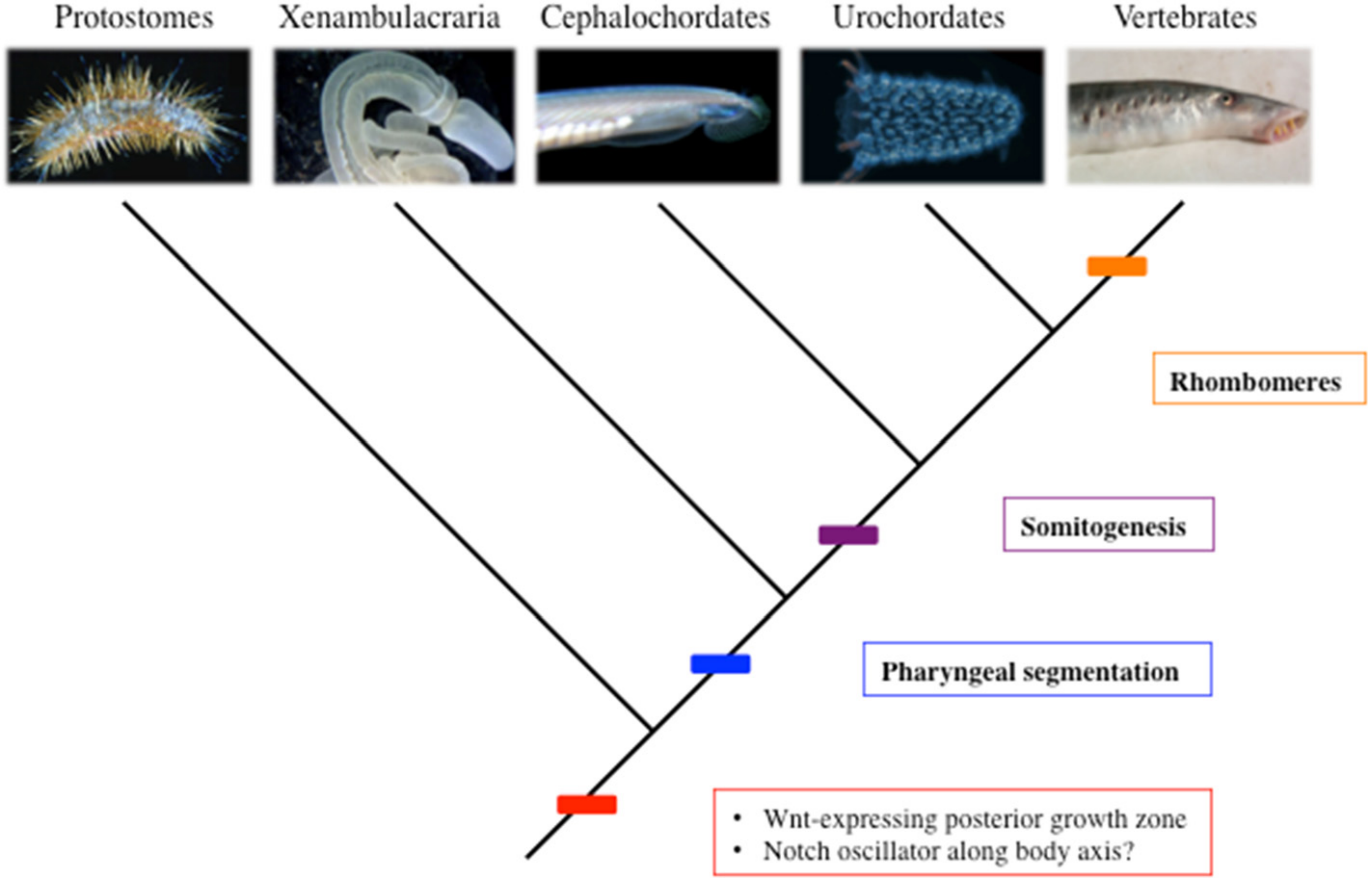
**The evolutionary history of segmentation in the vertebrate lineage.** Three instances of segmentation are found in extant vertebrates that are conserved with different invertebrate groups. While the ancestor of all Bilateria probably developed with a posterior growth zone expressing Wnts, and perhaps oscillating Notch signalling along the A/P axis, overt morphological segmentation appears later in the phylogeny. Pharyngeal segmentation can be dated to the deuterostome ancestor, while somitogenesis dates to the chordate ancestor and rhombomeric organisation of the hindbrain to the vertebrate stem.

An important point that emerges from this is that there was no ancestral process of segmentation that was co-opted by each of these processes. The three segmental systems of the vertebrates each arose *de novo* at different points during evolution (Figure 2). The most ancient is pharyngeal segmentation, and that is a feature of the deuterostomes, with somitogenesis following with the emergence of the chordates and finally rhombomere formation and the evolution of the vertebrates.

### Single or manifold evolutionary origins of segmentation?

There have been many discussions as to the evolutionary origin(s) of segmentation and there are two key issues here that must be confronted. The first is whether or not there is any evidence to support homology between the manifestations of segmentation seen in vertebrates with those displayed by other bilaterian clades. Attempts to homologize between segmentation in vertebrates and that seen in arthropods and annelids, have been strongly affected by their time. In the late 1980s and early 1990s comparisons were invariably drawn between the mechanisms underpinning the segmentation of the hindbrain and those directing segmentation in Drosophila. Both involved specification via transcription factor hierarchies and both resulted in the formation of lineage restricted compartments. However, as our molecular understanding of somitogenesis advanced it became more common to draw comparisons between that process and other modes of arthropod segmentation. For example, it was noted that segmentation in spiders involves Notch and Delta signalling [59]. Yet, for both of these comparisons, our extensive knowledge of the developmental processes underpinning rhombomere formation and somitogenesis would indicate that the highlighted commonalities are but simply superficial similarities.The mechanisms underpinning the segmentation of the Drosophila embryo are quite different from those in vertebrates. Drosophila segments are formed within a syncytium by a transcription factor cascade. While rhombomeres are formed via cell sorting, using Eph-ephrin signalling, lying downstream of a very different system of signalling molecules and transcriptional effectors. Furthermore, rhombomeres evolved with the vertebrates (Figure 2); they are clearly lacking in other deuterostomes, and thus it is unlikely that they could have a common origin with the segmental patterning systems of arthropods.

The formation of somites and the segments of many arthropods do share the characteristic that they are generated sequentially and that this is tied to axis elongation. But, as such, some of the shared features associated with somite formation and arthropod segmentation may indicate more general conserved bilaterian features, such as the involvement of a posterior wnt-secreting growth zone [60,61]. It is also very possible that the association between notch signalling and segments is the result of this very ancient signalling pathway becoming subservient to the segmentation processes in animals that organise their body plan in such a manner, and indeed as we have pointed out Notch signalling is not required for somite formation in vertebrates. Finally, it should be stressed again that somites are a chordate feature (Figure 2) and are not found in other deuterostomes and thus it seems again unlikely that somitogenesis could have a common origin with any mode of arthropod segmentation.

The second issue is the relative paucity of segmentation within the bilateria and its implications. As Hannibal and Patel point out, if segmentation is difficult to evolve, this would suggest that it had a single origin but that it was subsequently lost by the great majority of animal phyla and only retained in a few [2]. One conclusion following from this hypothesis would be that segmentation is readily dispensable in the generation of a functional body plan. By contrast, if segmentation is relatively easy to evolve then one would expect to observe unrelated, non-homologous, instances of segmentation in different phyla. Our discussion of the segmented systems of vertebrates would point us towards the latter option. We find that there is no single process of segmentation and, that in the lineage leading to the vertebrates, segmented structures evolved at least three times independently, in different germ layers and using different mechanics, at least three times. Thus it would seem that it is relatively easy to evolve segmentation.

## Conclusions

Hannibal and Patel make the excellent point that there is no merit in talking about segmentation without being explicit about what is being discussed. Thus with regards to segmentation in vertebrates, it is unhelpful to talk generally of segmentation and to lump together the processes of somitogenesis, rhombomere formation and pharyngeal arch development; these are chalk and cheese comparisons. It is more correct and useful to discuss how somites form, how rhombomeres emerge and how pharyngeal arches are generated. It is likewise uninformative and potentially confounding to talk of individual taxa as ‘segmented’ or ‘unsegmented’, or even ‘pseudosegmented’; it is much more sensible to talk about segmented structures or body regions individually.

Furthermore, as Hannibal and Patel note, it is incredibly difficult to arrive at a precise definition of segmentation and we would argue that this is because there is no single process of segmentation. Consequently, all definitions of segmentation are superficial; that is, repetition of structures along the main body axis - there is nothing deeper to be indicated. An analogous situation is that of wings - what is a wing? It is a structure that allows an animal to fly. Wings are a feature of flies, birds and bats but the definition of a wing has to be superficial because it describes non-homologous structures. Thus, many of the problems that arise with the concept of segmentation, and that we have discussed here, ultimately reflect a problem of terminology. The names that we apply to biological processes do not necessarily indicate anything beyond being useful appellations. Of course this is the problem of homoplasy and the only route to resolving this is to map any given biological process to the phylogeny.

## Competing interests

The authors declare that they have no competing interests.

## Authors’ contributions

This article was written by all four authors. All authors read and approved the final manuscript.
